# Better Knee, Better Me™: effectiveness of two scalable health care interventions supporting self-management for knee osteoarthritis – protocol for a randomized controlled trial

**DOI:** 10.1186/s12891-020-3166-z

**Published:** 2020-03-12

**Authors:** Kim L. Bennell, Catherine Keating, Belinda J. Lawford, Alexander J. Kimp, Thorlene Egerton, Courtney Brown, Jessica Kasza, Libby Spiers, Joseph Proietto, Priya Sumithran, Jonathan G. Quicke, Rana S. Hinman, Better Knee, Better Me™ study team, Anthony Harris, Andrew M. Briggs, Carolyn Page, Peter F. Choong, Michelle M. Dowsey, Francis Keefe, Christine Rini

**Affiliations:** 1 Centre for Health, Exercise and Sports Medicine, Department of Physiotherapy, School of Health Sciences, The University of Melbourne, Parkville, Melbourne, VIC 3010 Australia; 2 Medibank Private, Melbourne, VIC Australia; 3 Department of Epidemiology and Preventive Medicine, Monash University, Melbourne, VIC Australia; 4 Department of Medicine, The University of Melbourne, Melbourne, VIC Australia; 5 Research Institute for Primary Care and Health Sciences, Keele University, Keele, UK; 6 Centre for Health Economics, Monash University, Melbourne, VIC Australia; 7 School of Physiotherapy and Exercise Science, Curtin University, Perth, WA Australia; 8 St Vincent’s Hospital, Melbourne, VIC Australia; 9 Department of Surgery, St Vincent’s Hospital, University of Melbourne, Melbourne, VIC Australia; 10 Duke Pain Prevention and Treatment Research Program, Durham, North Carolina USA; 11 Hackensack University Medical Center and Georgetown University School of Medicine, Washington, USA

**Keywords:** Osteoarthritis, Exercise, Telerehabilitation, Weight management, Ketogenic diet, Knee, Pain, Obesity, RCT, Physiotherapy, Dietitian

## Abstract

**Background:**

Although education, exercise, and weight loss are recommended for management of knee osteoarthritis, the additional benefits of incorporating weight loss strategies into exercise interventions have not been well investigated. The aim of this study is to compare, in a private health insurance setting, the clinical- and cost-effectiveness of a remotely-delivered, evidence- and theory-informed, behaviour change intervention targeting exercise and self-management (*Exercise* intervention), with the same intervention plus active weight management (*Exercise plus weight management* intervention), and with an information-only control group for people with knee osteoarthritis who are overweight or obese.

**Methods:**

Three-arm, pragmatic parallel-design randomised controlled trial involving 415 people aged ≥45 and ≤ 80 years, with body mass index ≥28 kg/m^2^ and < 41 kg/m^2^ and painful knee osteoarthritis. Recruitment is Australia-wide amongst Medibank private health insurance members. All three groups receive access to a bespoke website containing information about osteoarthritis and self-management. Participants in the *Exercise* group also receive six consultations with a physiotherapist via videoconferencing over 6 months, including prescription of a strengthening exercise and physical activity program, advice about management, and additional educational resources. The *Exercise plus weight management* group receive six consultations with a dietitian via videoconferencing over 6 months, which include a very low calorie ketogenic diet with meal replacements and resources to support behaviour change, in addition to the interventions of the *Exercise* group. Outcomes are measured at baseline, 6 and 12 months. Primary outcomes are self-reported knee pain and physical function at 6 months. Secondary outcomes include weight, physical activity levels, quality of life, global rating of change, satisfaction with care, knee surgery and/or appointments with an orthopaedic surgeon, and willingness to undergo surgery. Additional measures include adherence, adverse events, self-efficacy, and perceived usefulness of intervention components. Cost-effectiveness of each intervention will also be assessed.

**Discussion:**

This pragmatic study will determine whether a scalable remotely-delivered service combining weight management with exercise is more effective than a service with exercise alone, and with both compared to an information-only control group. Findings will inform development and implementation of future remotely-delivered services for people with knee osteoarthritis.

**Trial registration:**

Australian New Zealand Clinical Trials Registry: ACTRN12618000930280 (01/06/2018).

## Background

Knee osteoarthritis (OA) is a major public health problem [1] and incurs enormous indirect and direct healthcare costs [2]. All current clinical guidelines recommend non-surgical non-drug treatments for first-line management of OA, including education/advice, exercise, and, if appropriate, weight loss [3, 4]. However, there is evidence that a substantial proportion of people with OA in Australia are not receiving recommended care [5]. For example, although surgery is only recommended for those with severe symptomatic OA who do not respond to conservative management approaches, data from an Australian hospital found that 33% of people referred for orthopaedic management had not engaged in any non-drug conservative management methods [6]. From 2005 to 06 to 2015–16, Australia has seen a 38% rise in the rate of total knee replacements for OA [7], yet 20% of people undergoing knee replacement surgery report unsatisfactory outcomes [8]. Given that the prevalence of knee OA is projected to rise in the coming decades [2, 9], there is an urgent need to increase uptake of recommended conservative management options through implementation of effective, accessible and scalable models of service delivery.

The benefits of therapeutic exercise for people with knee OA are well-established [10], with similar efficacy to analgesics and non-steroidal anti-inflammatory drugs, but with fewer side-effects [11, 12], and with fewer risks than joint replacement surgery. Given that muscle weakness is common amongst people with OA [13], muscular strengthening exercises are important and can help reduce pain, and improve physical function [14]. In addition, as most people with OA do not meet physical activity recommendations [15], advice to increase physical activity is also important. For example, research has demonstrated that people with knee OA who are less sedentary have better physical function, independent of time spent doing moderate or vigorous physical activity [16, 17], and that a threshold of 6000 steps per day discriminates between people with knee OA who do and do not develop functional limitations 2 years later [18].

Given that people with OA who are overweight or obese tend to experience more severe symptoms [19, 20] and are more likely to undergo joint replacement surgery [21], weight loss is an important component of management. However, although weight loss is recommended by evidence-based guidelines [4, 22, 23] there is only limited evidence to support its efficacy in improving symptoms of pain and dysfunction. In the literature, interventions often combine both exercise and weight loss components, making it difficult to identify the independent effects of weight loss on outcomes. One pivotal trial involving 454 overweight and obese adults with knee OA investigated the effects of an intensive diet program, with and without exercise, conducted in a rigorously monitored environment [24]. The combined diet and exercise group lost approximately 10% of body weight, compared to 2% in the exercise only group, leading to reduced knee loads and inflammation compared to the exercise only group. While statistically significant benefits on pain (1-point difference (95% confidence interval: 0.3 to 1.7) on 20-point scale) and function (4.3-point difference (2.1 to 6.5) on 68-point scale) were also seen, the magnitude of these benefits are small and may be of questionable clinical importance. Furthermore, although the diet and exercise intervention was reported to be more cost-effective than usual care (pharmacological NSAIDs regimen followed by total knee replacement surgery) [25], the cost-effectiveness analyses did not include data from the exercise only group. Thus, it remains unknown whether a combined intervention of diet and exercise is more cost-effective than exercise alone.

Although the findings of Messier and colleagues [24] support the efficacy of combined weight loss and exercise programs, their interventions were time-intensive and potentially costly, with the weight loss component involving 12 individual and 42 group sessions for nutrition education and behavioural support over 18-months, and the exercise component involving three group exercise sessions per week for 6-months. Given that many barriers to self-management of OA relate to inaccessibility and/or costs of healthcare [26, 27], implementation of such an intensive program outside of a research setting is likely to be difficult in most community or hospital settings, either public or private, without considerable cost and time burdens. Identifying effective programs that minimise active involvement by clinicians is therefore important. One way in which to improve the accessibility and scalability of care is to provide it remotely via technology (telerehabilitation), allowing patients to consult from their own home or workplace. There is emerging evidence that telerehabilitation for people with musculoskeletal conditions improves pain and function and is equivalent to outcomes following traditional in-person consultations [28]. More recently, there is evidence that exercise advice and prescription delivered by physiotherapists via videoconferencing leads to improvements in pain and function in people with knee OA [29]. Importantly, such a service is also acceptable to both the people with knee pain receiving care and the physiotherapists delivering it [30]. Similarly, dietary interventions for weight loss can also be effective when delivered remotely. A recent systematic review and meta-analysis found that dietary interventions for people with chronic diseases (i.e. obesity, diabetes, heart disease, hypertension, stroke, or kidney disease) delivered by dietitians or nurses via video or telephone improved diet quality and led to significant weight loss and reduced waist circumference [31]. No previous studies have investigated the effectiveness of remotely-delivered exercise and weight management programs by physiotherapists and dietitians for people with knee OA who are overweight or obese.

For many people with OA, undertaking an exercise and/or weight management program requires considerable changes in lifestyle behaviours over prolonged periods of time. As such, it is important that intervention development is underpinned by behaviour change theory, and informed by best practice in chronic disease management and self-management support programs. Interventions must include provision of high quality information. However, education alone is insufficient to support behavioural change. Healthcare clinicians must also have the communication and psychosocial skills necessary to facilitate long-term changes in behaviour [32, 33]. Incorporating behavioural counselling [34, 35] and specific behaviour change techniques that are effective for supporting change in exercise and eating behaviours in this population [36] into intervention design will facilitate participants to achieve and sustain effective self-management practices in the long-term.

The aim of this study, conducted in a private health insurance setting, is to compare the effectiveness of two remotely-delivered, evidence- and theory-informed, behaviour change interventions to provide information and behaviour change support for i) exercise and; ii) exercise plus weight management, to each other, and to an information-only control for people with knee OA who are overweight or obese. We hypothesise that the exercise plus weight management intervention will lead to greater improvements in pain and function than the exercise intervention and that both interventions will be more effective than information only.

## Methods/design

### Design

**Fig. 1:**
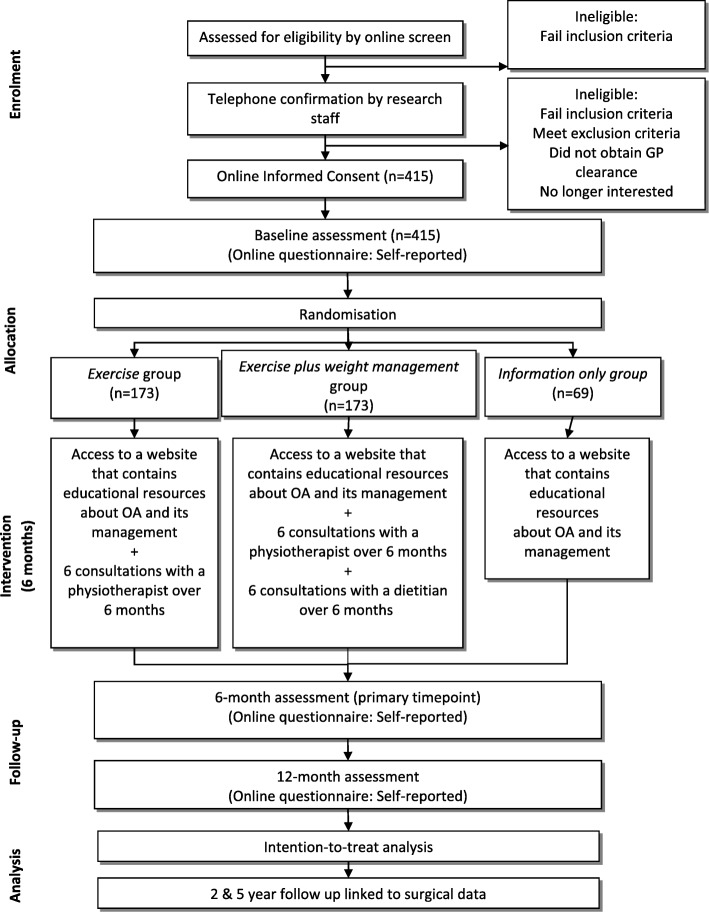
Participant flow through the randomized controlled trial

The Better Knee, Better Me™ trial is a three-arm pragmatic superiority parallel-design randomised controlled trial. Figure 1 outlines the RCT phases. This protocol has been developed according to the SPIRIT statement [37]. The trial has been prospectively registered in the Australian New Zealand Clinical Trials Registry (ACTRN12618000930280). The trial is conducted at The University of Melbourne. The effectiveness and cost-effectiveness of interventions will be assessed by analysing a range of outcomes and reported as the main trial results. Nested qualitative studies of participants’ and clinicians’ experiences participating in the trial will be used to explore the acceptability and usefulness of the services, as well as explore the barriers and facilitators to engagement. However, this will be reported separately to the main trial results.

### Participants

A total of 415 participants who have private health insurance with Medibank Private at a level that includes cover for arthroplasty surgery are recruited from around Australia. Medibank Private is one of Australia’s largest health funds, with 3.7 million members across Australia [38]. Inclusion and exclusion criteria are outlined in Table 1. Medibank Private send targeted invitations, predominantly via email, to potentially eligible members. They also advertise the study in their member newsletters and on their website. The University of Melbourne manage the clinical screening of volunteers. Screening occurs via a two-step process; i) via an online survey and, if eligible, ii) via additional telephone screening by research personnel at The University of Melbourne. Additional clearance to participate is sought from a general practitioner for anyone who does not pass pre-exercise screening questions [40], reports more than 1 fall in the past 12 months, or is house-bound due to mobility limitations (e.g. concurrent disabling low back pain).

**Table 1:** Participant eligibility criteria

Inclusion criteria	Exclusion criteria
Fulfil National Institute for Health and Care Excellence [3] clinical criteria for osteoarthritis: • Age > 45 years; • Have activity related joint pain; and • Have morning stiffness ≤30 min. Average knee pain severity ≥4 on 11-point numeric rating scale (NRS, where 0 = no pain, 10 = worst pain possible) in the past week History of knee pain on most days for at least 3 months Aged < 81 years – due to potential safety reasons and additional co-morbidities Body mass index (BMI) ≥ 28 kg/m^2^ and < 41 kg/m^2^. The lower BMI limit was chosen according to recommendations [39] that VLCD are effective in supporting weight loss in people with a BMI > 27. The upper limit was chosen as these individuals often require more extensive intervention and for possible safety reasons Member of Medibank with a level of cover that includes arthroplasty surgery Able to give informed consent and to participate fully in the interventions and assessment procedures Willing to follow advice for self-management, participate in exercise/physical activity and/or participate in a weight loss program if part of their treatment program Have the ability to weigh themselves (e.g. have access to the same set of bathroom scales)	Booked for knee surgery on either knee Have had all eligible knee joints replaced by arthroplasty Knee surgery within the past 6 months Unable to speak or read English Self-reported diagnosis of rheumatoid arthritis or other inflammatory arthritis Other medical condition or upcoming medical procedures that in the opinion of the research staff and/or investigators would preclude participation Private health insurance claims related to cancer treatment or inpatient neurological rehabilitation in the previous 12 months, palliative care, or acquired brain injury Unable to use/access a telephone and internet For those identified as at risk from the pre-exercise and falls screening, doctor does not give clearance Used low-calorie meal replacement products (e.g. Optifast/Optislim) for weight loss in previous 6 months Currently, or in the past 6 months, undertaking regular strengthening exercises for the knee Unable to undertake VLCD for medical reasons including: i. Self-reported diagnosis of Type 1 diabetes ii. Self-reported Type 2 diabetes requiring insulin or other medication apart from metformin iii. Self-reported warfarin use iv. Stroke or cardiac event in previous 6 months v. Unstable heart condition vi. Fluid intake restriction

*BMI* Body mass index, *VLCD* Very low calorie diet

### Data collection and management

Data are collected via online questionnaire and stored on a secure password-protected server. The “study knee” is the painful knee or, in people with bilaterally eligible knees, the most painful knee.

### Randomisation allocation concealment and blinding

The randomisation schedule is computer generated and prepared by the study biostatistician. Following completion of the baseline questionnaire, participants are randomly allocated to either the *Exercise* group, the *Exercise plus weight management* group, or the information only (*Control*) group. Due to the differences expected to be observed between treatment groups (effect size of 0.3) and between the least-performing treatment group and the control group (effect size of 0.4), to minimise the number of control group participants required, we will recruit fewer participants in the control group than in either treatment group: see details in the Trial sample size section below. This leads to a randomisation ratio of 5:5:2 (for every 5 participants randomised to a treatment group, we will only randomise 2 to the control group). We expect to see a smaller difference between the two treatment groups than between the least-performing treatment group and the control group. Randomisation will be stratified by history of knee surgery (arthroscopy or contralateral arthroplasty). Participants allocated to the *Exercise* group are randomly allocated to one of three physiotherapists. Participants allocated to the *Exercise plus weight management* group are randomly allocated to one of the same three physiotherapists as in the *Exercise* group, and one of five dietitians. If a physiotherapist or dietitian is unavailable (e.g. sick, on holidays), participants are re-randomised to another available clinician. To conceal group allocation, a researcher not involved in participant recruitment accesses the randomisation schedule via a password-protected computer program.

Given that all primary and secondary outcomes are self-reported, participants are also the assessors. Participants are blinded to study hypotheses, however the components of each treatment arm are disclosed during recruitment. This replicates real-world conditions whereby consumers are fully informed about the nature of the treatment components before choosing whether or not to participate/receive an intervention. Physiotherapists and dietitians are not blinded to group allocation.

### Information only (control) group

The information only (*Control*) group receive information, advice, and education about OA and its management via a bespoke website developed by the research team which is only accessible via password login. The website includes: i) educational information including understanding OA, recommended treatment options (including a total knee replacement decision aid), exercise and physical activity, weight loss, understanding and managing pain (including audio files to facilitate mini-relaxations and pleasant imagery), sleep, and “success stories” from other people with knee OA, and; ii) links to recommended external websites for further help and support (e.g. MyJointPain, painHEALTH, Musculoskeletal Australia). Participants have access to the study website from enrolment to completion of the 12-month follow-up questionnaire.

### Intervention design

Design of the interventions is underpinned by the Chronic Care model [41], recommendations for design and evaluation of self-management support programs [33], and the information-motivation-behavioural skills theoretical model (IMB) for behaviour change [42, 43]. The Chronic Care model [41] explains the importance of productive interactions between clinicians with the right skills and patients who are informed and activated. An intervention with the aim of facilitating effective self-management by patients must both activate and then support patient behaviour change. The IMB model contends that although information is a pre-requisite for change, on its own information will not activate change [44]. The model explains that having the motivation to change, and the behavioural skills needed in order to change, are independent and essential determinants [43]. Other interventions based on this model have been effective in influencing behavioural change across a variety of clinical applications [45]. This model of change is consistent with the recommendation that in order to be effective, self-management support programs must include motivational coaching interventions as well as educational interventions that link knowledge with skills [33].

Two recent systematic reviews support the effectiveness of coaching interventions, such as Motivational Interviewing, for managing chronic conditions [35] and chronic painful musculoskeletal conditions [34], specifically in regard to increasing physical activity behaviours. Coaching from clinicians must be patient-centred and tailored to the needs and concerns of the individual [33]. Training and protocols for the clinicians are important intervention components, especially since these coaching skills are not often taught during entry to practice training for health professionals and both clinicians and trainees report skills deficits in this area [46]. In addition to being able to effectively deliver high quality information to facilitate desired behaviour change, clinicians also need to incorporate specific behaviour change techniques such as shared decision-making, goal setting, problem solving, confidence building, review and reinforcement. Additional techniques that have been shown to be effective for supporting change in exercise and eating behaviours and that are included in the interventions include providing information about the consequences of the behaviour, encouraging social support, phased action planning, self-monitoring, graded tasks, reinforcing successful behaviour, prompting focus on past success, providing feedback about the participant’s behaviour, barrier identification, and relapse prevention/coping planning [36].

**Fig. 2:**
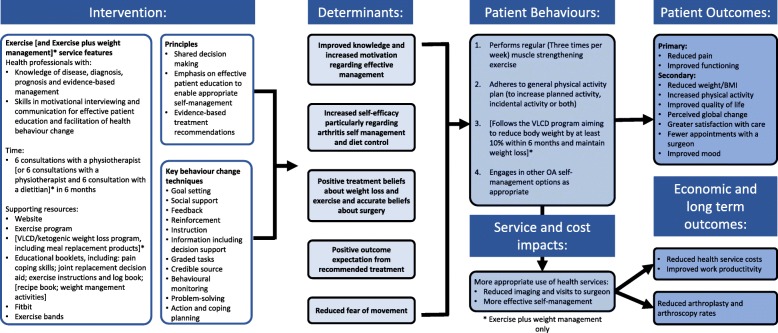
Logic model depicting the rationale underpinning the *Exercise* and *Exercise plus weight management* models of service delivery *Exercise plus weight management only VLCD: very low calorie diet

The intervention design is additionally based on best practice recommendations for management of knee OA [3, 4] and obesity [39, 47], plus exercise and weight loss interventions with proven effectiveness. Stakeholder input was sought from our key collaborators: Medibank Private and the Weight Control Clinic at Austin Health as well as our multidisciplinary and cross-sectoral research group. Consumers provided input into various aspects of the trial including the resources and study name. The rationale for the interventions is depicted in a program logic model in Fig. 2.

All potential participants are provided with a plain language statement which describes what is involved in each of the study groups and the level of commitment required. Participants are reminded of this during the online and telephone screening process, and are excluded if they feel they are not able to commit fully to either of the three study groups.

### Exercise intervention

The *Exercise* intervention consists of educational information and links to external websites as for the *Control* group (bespoke website, in addition to exercise modules related only to this intervention) plus six individual consultations with a physiotherapist via videoconferencing over the internet (Zoom Video Communications Inc., California USA) over 6-months (Table 2). The physiotherapist consultations include individualised advice on treatment options, decision support, a structured exercise and physical activity plan, behaviour change support, and facilitation of other self-management strategies including pain coping skills training. Educational resources (Table 2) are provided in hardcopy via post and electronically via the study website. Participants are also posted three exercise resistance bands (green, red, and blue) for strengthening exercises and a Fitbit (Flex 2 model) to help track and monitor physical activity.

**Table 2:** Summary of resources provided to participants in the *Exercise* and *Exercise plus weight management* groups

Resource	Description of content/purpose	***Exercise*** group	***Exercise plus weight management*** group
Study website	Information about OA, treatment options, exercise and physical activity, weight loss, managing pain, sleep, and stories from other people with knee OA	√	√
Consultations with a physiotherapist	6 video consultations over 6-months. Advice on treatment options, structured exercise and physical activity plan, and behaviour change support	√	√
Consultations with a dietitian	6 video consultations over 6-months. Helps participant undertake VLCD with behaviour change support		√
Exercise bands	3 exercise resistance bands (green, red, and blue) for strengthening exercises	√	√
Activity tracker (Fitbit)	To help track and monitor physical activity	√	√
Plastic portion plate	To help manage portion sizes		√
Optifast® meal replacements	6-months’ worth of meal replacements for the VLCD		√
Educational video about the VLCD	Short video about the VLCD featuring endocrinologists and dietitian experts, and a person with knee OA		√
**Booklets**			
Preparing for your consultations	Information about consultations, instructions on how to use Zoom videoconferencing, and Fitbit instructions	√	√
Osteoarthritis information	Understanding knee OA, common management options, weight loss, pain coping skills, and sleep	√	√
Exercise booklet	Strengthening exercise instructions and photos	√	√
Knee care plan and exercise log book	Templates to record details of management plans and completed exercises	√	√
Knee replacement surgery for osteoarthritis-related pain	Decision aid about joint replacement surgery, including the benefits and harms of surgery	√	√
Weight management ‘how to’ guide	Describes the VLCD and provides information about healthy food choices and portion sizes		√
Weight management behavioural support activities	Workbook containing information and templates to track weight, a food diary, tips to find a support person, identifying food triggers, planning for “at risk” situations, overcoming barriers, changing thought patterns, and monitoring hunger levels		√
Recipe book	Recipes that are suitable for the VLCD		√
Food list pocket guide	List of suitable low carbohydrate ingredients to consume when on the VLCD		√

*OA* Osteoarthritis, *VLCD* Very low calorie diet

Before their first consultation with the physiotherapist, participants complete a pre-consultation survey asking about their main problems and goals, a brief history of their knee symptoms, and other health problems. The initial physiotherapy consultation is approximately 45 min long, with follow-up consultations being approximately 20 min. Consultations are recommended to occur in weeks 1, 3, 7, 11, 16, and 21, but the precise timing is negotiated between each participant and their physiotherapist.

Together with the participant, the physiotherapist develops an individualised management plan aiming to include the following components: i) a structured and progressive muscle strengthening exercise program; ii) a tailored physical activity plan to increase incidental and general physical activity and reduce sedentary behaviour, including a daily step goal if agreed by the participant, and; iii) other practical self-management strategies (e.g. activity pacing, pain coping strategies, sleep hygiene). Physiotherapists use motivational interviewing principles and techniques to develop motivation (readiness to change) and self-efficacy as well as assist the participant to overcome barriers to enacting their agreed management plan. Education about OA is provided from a biopsychosocial perspective to facilitate confidence in ability to manage pain. For participants who have been identified during the baseline questionnaire as having a preference towards total joint replacement surgery in their knee, physiotherapists discuss the benefits and harms of surgery with participants. For those who are not yet considering surgery, information about the likelihood of future need for surgery is discussed. During follow-up consultations, physiotherapists review progress and reassess goals, making modifications as required.

For the strengthening exercise program, physiotherapists choose from the list of exercises shown in Table 3 (based on exercises used in previous trials by the Centre which were found to be effective [29, 48]) and aim to prescribe 5–6 exercises, including at least one from each category (i.e. quadriceps strengthening) and an optional extra. The exact number of exercises, as well as sets/repetitions, is negotiated between the physiotherapist and participant. Intensity is determined using a modified Rating of Perceived Exertion (RPE) [49], where it should feel “hard” to “very hard” to perform a full set of each exercises. Participants are encouraged to complete exercises three times per week. For the physical activity plan, physiotherapists encourage the participant to increase their general and incidental levels of physical/aerobic activity based on their individual needs and goals, as well as their current level of activity. Participants are encouraged to use the provided Fitbit to record and monitor their daily step count. Prescription of exercises/physical activity plans is based on the clinical history and functional ability of the participant. Participants are provided with a study email they can use to contact the clinicians between consultations if they have any questions or encounter any issues.

**Table 3:** Home exercise protocol

**Maximum of 6 exercises** (with progression as appropriate) 2 knee extensor strengthening exercises 1 hip abductor strengthening exercise 1 hamstring strengthening exercise 1 calf strengthening exercise 1 other exercise as appropriate			
**1. Quads strengthening (aim to include two exercises)**			
**Knee extension**	Non weight-bearing	Q1. Seated knee extension (with resistance) with 5 s hold	**Progression:** Increase with theraband resistance – red through to black
	Non weight-bearing	Q2. Inner range quads over roll (with resistance) with 5 s hold	**Instruction:** Put a rolled up towel under your arthritis knee. Keep the knee cap and toes pointing toward the roof. Keeping the back of the knee in contact with the towel, push the back of your knee down into the towel and straighten your arthritis leg
**Sit-to-stand**	Weight-bearing	Q3. Sit to stand without using hands	**Progression:** lower chair height, hover above the seat without touching down (3 s hold), add resistance band around knees and push outwards keeping knees over toes
	Weight-bearing	Q4. Sit to stand with more weight on involved knee	**Instruction:** Take more weight by either a) placing uninvolved further forward b) shift both feet sideways so study leg is midline
**Steps**	Weight-bearing	Q5. Step-ups	**Progression:** Increase step height
	Weight-bearing	Q6. Forward touchdowns from a step	**Progression:** Increase step height, don’t touch floor
	Weight-bearing	Q7. Step-ups with weight	**Instruction:** Hold 2 kg of weight either a) against chest, b) in each hand, c) in one hand while holding for balance, or d) in backpack **Progression:** Increase step height, increase weight
	Weight-bearing	Q8. Forward touchdowns from a step with weight	**Instruction:** Hold 2 kg of weight either a) against chest, b) in each hand, c) in one hand while holding for balance, or d) in backpack **Progression:** Increase step height, increase weight
**Wall squats**	Weight-bearing	Q9. Partial wall squats for 5 s hold	**Progression:** more weight on study side
	Weight-bearing	Q10. Split leg wall squats for 5 s hold	**Instruction:** Step feet away from wall (about 30 cm) and move non-involved leg a further 15 cm away from the wall.
**Controlled squats**	Weight-bearing	Q11. Controlled partial squat with 5 s hold	
**Leg Sliding**	Weight-bearing	Q12. Forward and backward sliding of uninvolved leg	**Instruction:** Keep weight on study leg. Concentrate on keeping knee positioned over the foot
	Weight-bearing	Q13. Forward and backward sliding of uninvolved leg with resistance band pulling study leg laterally	**Instruction:** Place loop of elastic band around involved knee and leg of a table to provide a pull outwards on knee that you must resist by keeping knee in line with foot. **Progression:** Increase with theraband resistance – red through to black
	Weight-bearing	Q16. Sideways sliding of uninvolved leg	**Instruction:** Keep weight on study leg. Concentrate on keeping knee positioned over the foot
	Weight-bearing	Q17. Sideways sliding of uninvolved leg with resistance band pulling study leg laterally	**Instruction:** Place loop of elastic band around study leg and leg of a table to provide a pull outwards on knee that you must resist by keeping knee in line with foot. **Progression:** Increase with theraband resistance – red through to black
**Step to single leg balance**	Weight-bearing	Q14. Step with study leg to about 30° knee flexion for single leg balance	**Instruction:** Take a step forwards with study leg, keeping knee bent to about 30° knee flexion. Allow uninvolved leg to lift off and practice balancing as long as you can
	Weight-bearing	Q15. Step with study leg to about 30° knee flexion for single leg balance with arm movements	**Instruction:** Take a step forwards with involved knee, keeping knee bent to about 30° knee flexion. Allow uninvolved leg to lift off and practice balancing as long as you can, while raising arms out to side and above head in an arc.
**2. Hip abductor strengthening (1 exercise)**			
**Standing hip abduction**	Weight-bearing	HA1. Side leg raises in standing with 5 s hold	**Progression**: Increase theraband resistance – red through to black, add another 5 s halfway
**Side stepping**	Weight-bearing	HA2. Crab walk with resistance band	**Progression:** Increase theraband resistance – red through to black
**Standing hip abduction**	Weight bearing	HA3. Wall push standing on study leg for 20 s	**Progression:** Hold weight in hand, increase the hold time
	Weight bearing	HA4. Wall push standing on study leg positioned up to 45° knee flexion	
**3. Hamstring strengthening (1 exercise)**			
**Bridging**	Weight-bearing	HG1. Bridge with 5 s hold	
	Weight-bearing	HG2. Split leg bridge with 5 s hold	**Instruction:** Place feet hip-width apart, then move study leg slightly closer toward your bottom and slightly in towards centre
	Weight-bearing	HG3. Single-leg bridge on study leg with 5 s hold	**Version A:** Lift bottom. Keeping hips level, lift uninvolved leg off the floor/bed. Hold for 5 s. Slowly lower uninvolved leg back to the floor. Then slowly lower bottom back to floor. **Version B:** Lift uninvolved knee off floor/bed. Lift bottom and take all weight through study leg. Hold for 5 s. Slowly lower your bottom back to floor/bed.
**Standing knee flexion**	Non weight-bearing	HG4. Hamstring curls -Standing over bench knee flexion with 5 s hold	
	Non weight-bearing	HG5. Hamstring curls -Standing over bench knee flexion with 5 s hold against resistance band	**Instruction:** Place one end of an elastic band securely around ankle of study knee. Place the other end of elastic around your opposite foot so you are standing on it.
**Seated knee flexion**	Non weight-bearing	HG6. Seated knee flexion	**Instruction:** Place one end of an elastic band securely around a stable object (e.g. a heavy table leg). Loop the other end around the ankle of your study leg. Keeping your opposite foot on the floor, pull against the elastic band and bend your knee more. **Progression:** Increase with theraband resistance – red through to black
**Standing hip extension**	Non weight-bearing	HG7. Hip extension with knee bent (90°) - standing over bench with 5 s hold	
	Non weight-bearing	HG8. Hip extension with knee straight - standing over bench with 5 s hold	
	Non weight-bearing	HG9. Hip extension with knee straight with resistance band - standing over bench with 5 s hold	**Instruction:** Place one end of an elastic band securely around ankle of study knee. Place the other end of elastic around your opposite foot so you are standing on it.
**4. Calf strengthening (1 exercise)**			
**Standing plantar-flexion**	Weight-bearing	C1. Double heel raises with 5 s hold	
	Weight-bearing	C2. Single heel raises with 5 s hold	
	Weight-bearing	C3. Double heel raises with 5 s hold over edge of step	
	Weight-bearing	C4. Double heel raises with 5 s hold over edge of step	
**5. Arm strengthening (if appropriate)**			
**Bicep curls**	Non Weight- bearing	1. Bicep curls	**Progression:** increase resistance
**Wall push ups**	Weight- bearing	2. Wall push ups	**Progression:** increase repetitions

During each consultation, physiotherapists complete online treatment notes recording the call duration, topics discussed, clinical history, personal motivators, and details of the participant’s management plan.

### Exercise plus weight management intervention

Participants who are allocated to the *Exercise plus weight management* intervention receive all components of the *Exercise* intervention plus 6 individual videoconferencing consultations with a dietitian (over 6 months) who helps the participant undertake a weight management program involving a ketogenic very low calorie diet (VLCD) [50]. This diet was chosen as it has been shown to lead to greater weight loss in the short-term when compared to low-fat diets [51, 52] and the release of ketones, produced by the liver during fatty acid oxidation while fasting or on a diet that restricts carbohydrates, can help suppress appetite and contribute to further weight loss [50]. Participants are provided with Optifast® meal replacements (Nestlé Health Science, Rhodes, Australia) (or Optislim® [OptiPharm Pty Ltd., Clayton, Australia] if unavailable or the participant is vegetarian) and additional educational resources to support their weight loss (Table 2). Participants are encouraged to lose at least 10% or more of their body weight, as this has been associated with clinically important improvements in pain [53, 54].

In addition to the five educational booklets provided in the *Exercise* intervention, participants in the *Exercise plus weight management* intervention are provided with four additional booklets (described in Table 2). Participants are also mailed a plastic “portion plate” (https://www.nestle.com.au/nhw/nestle-portion-plates) to help them manage meal portion sizes. A short educational video about the VLCD featuring endocrinologists and dietitian experts, and a person with knee OA, is also available on the study website.

Participants’ first consultation with the dietitian takes place within the same week as their first physiotherapist consultation and is approximately 45 min in duration, with follow-up consultations approximately 20 min. During the initial consultation, appropriate weight loss goals, including a target weight, and a management plan are developed. Participants are encouraged to commence the VLCD, however they are also able to commence a modified version of the diet (e.g. one meal replacement only) if deemed necessary (e.g. if they do not like the meal replacements or are having difficulty adhering). Participants are asked to weigh themselves weekly and record their progress in their log book. During subsequent consultations, dietitians discuss progress and use motivational interviewing principles and techniques to assist motivation [35], self-efficacy, and overcoming obstacles to completing the agreed self-management plan. Dietitians also guide participants through the resource booklets provided, including activities to help them adhere to their self-management plan such as planning for unforeseen events (e.g. eating out) and choosing a support person. Once the participant has reached their target weight, they can choose whether to transition to a weight maintenance phase or continue with the VLCD program and aim for further weight loss.

The VLCD involves replacing two meals, generally breakfast and lunch, with meal replacements. Very low calorie diet meal replacement products are formulated to provide the adequate micronutrients (vitamins, minerals, and metals), and come as bars, shakes, or soups in a variety of flavours. On the diet, one meal (generally dinner) comprises protein (e.g. white or red meat, fish or seafood, eggs, or tofu) and non-starchy vegetables/salad. A small amount (i.e. 1 tablespoon) of oil/fat is also recommended for this meal to reduce the risk of gallstone formation. In total, the diet contains approximately 800 cal (3280 kJ).

Transitioning off the VLCD (after target weight is reached) is done by reintroducing foods containing carbohydrates and moving to only one meal replacement per day. This transition phase usually lasts at least 2 weeks, after which participants commence a healthy eating diet which is consistent with the principles of the Commonwealth Scientific and Industrial Research Organisation (CSIRO) total wellbeing diet [55] (i.e. high protein, low glycaemic index carbohydrate, low fat). Participants are encouraged to weigh themselves regularly thereafter (e.g. once per week). Participants who regain 2 kg or more are advised to restart the VLCD with meal replacements for 1–2 weeks.

Dietitians complete online consultation notes using the same platform as physiotherapists, where they are able to record details about agreed weight management plans and barriers/motivators that have been discussed, as well as record which resources/activities participants were asked to read/complete. Dietitians and physiotherapists are also encouraged to communicate with each other through their consultation notes (e.g. to flag any important issues that the participant might be having). To reduce the risk of loss of muscle mass during their weight management program, physiotherapists also recommend that participants in this group complete regular arm strengthening exercises (Table 3) in addition to their leg strengthening program. Participants are provided with a study email they can use to contact the clinicians between consultations if they have any questions or encounter any issues.

### Physiotherapist and dietitian training

Three musculoskeletal physiotherapists are employed to deliver the intervention. Physiotherapist selection criteria includes having experience managing people with chronic diseases, specifically knee OA, and current registration with the Australian Health Practitioner Regulation Agency. Five accredited dietitians, with at least 2 years of clinical experience and some experience assisting people with weight loss, are employed to deliver the weight management intervention. Prior to the start of the trial, clinicians complete training in:

i) best-practice OA management: dietitians attend a half day workshop in evidence-based management of OA, delivered by members of the research team. Briefly, this includes an introduction to the purpose of the trial, OA definition, diagnosis, risk factors and prognosis, evidence-based practice (including education, exercise therapy, weight loss, and psychological treatments), and principles of shared decision-making and health behaviour change techniques.
ii) motivational interviewing skills: dietitians and physiotherapists attend a 2-day training course from Health & Wellbeing Training Consultants, who specialise in training clinicians in motivational interviewing. Case studies presented during trained are designed to replicate potential scenarios anticipated to arise during the trial. Following these first two training days, clinicians conduct an initial and follow-up video consultation with a practice participant (one per clinician) to practice using their motivational interviewing skills and recording consultation notes. Practice consultations are audio recorded and audited for fidelity by a member of the research team using the Motivational Interviewing Treatment Integrity tool (MITI 4.2.1). Group level feedback is provided to clinicians at a half day workshop approximately 1 month after the initial two training days;
iii) VLCD: dietitians attend a 1-h webinar delivered by the research team on the specifics of the VLCD. Physiotherapists are asked to watch the video recording of the presentation.
iv) study-specific protocols: dietitians and physiotherapists attend a 1–2 h session (supplemented by detailed study treatment manuals) to ensure clinicians were familiar with all study procedures and processes. All clinicians are provided with a hardcopy of the study manual as well as hardcopies of all participant booklets to aid discussions during consultations.

### Treatment fidelity

After the trial commences, clinicians are able to discuss with the research team via phone or email any issues with delivering the intervention as planned, including using the motivational interviewing principles. Regular meetings are held with all clinicians and the trial team to discuss any issues or provide feedback. All consultations are audio-recorded and a random subset will be audited for fidelity using the MITI 4.2.1. Fidelity is also evaluated by auditing the electronic treatment notes, which includes details of participant management plans (e.g. the exercises prescribed and dietary plans).

### Dealing with adverse events

Expected adverse events include: i) transient increase in knee pain or swelling due to increased exercise or physical activity; ii) feeling hungry, fatigued, fuzzy-headed, and/or having headaches, and either diarrhoea or constipation during the first week after commencing the VLCD. Participants are advised to report any adverse events to the study coordinator as soon as they can, which will be documented in treatments notes and an adverse events log. If necessary, treatment is discontinued and further medical advice is arranged. Adverse events are also collected via self-report in the 6- and 12-month questionnaires.

Clinicians are instructed to report any adverse events to the trial coordinator, record the event in their consultation notes, and refer participants to their general practitioner if necessary. Participants are provided with a “help” email address to contact if they have any issues with their exercise/physical activity or weight management program in between consultations. The email is monitored daily by research staff and, if necessary, staff can seek further advice/guidance from the participant’s physiotherapist and/or dietitian.

### Outcomes

Outcome measures and time points are described in Table 4. Our primary and secondary outcome measures are recommended and validated for knee OA trials [56, 57] and are measured at baseline, 6 and 12 months.

**Table 4:** Summary of measurements to be taken

Domain	Data collection instrument	Time points		
		Baseline	6 M	12 M
**Descriptive data**				
Age, gender, height, body mass index		✓		
Duration of knee OA symptoms		✓		
Previous treatments and surgery		✓		
Problems in other joints		✓		
Medical history		✓		
Expectation of treatment outcome	5-point ordinal scale	✓		
**Primary outcome measures**				
Average knee pain in past week	11-point NRS	✓	✓	✓
Physical function in past 48 h	WOMAC physical function subscale	✓	✓	✓
**Secondary outcome measures**				
Weight	Self-reported	✓	✓	✓
Physical activity	IPEQ-W questionnaire	✓	✓	✓
Health-related quality of life	AQoL-8D questionnaire	✓	✓	✓
Perceived change since baseline	Overall change, 7-point ordinal scale		✓	✓
	Change in pain, 7-point ordinal scale		✓	✓
	Change in function, 7-point ordinal scale		✓	✓
	Change in physical activity, 7-point ordinal scale		✓	✓
Satisfaction with care	7-point ordinal scale		✓	✓
Appointment with orthopaedic surgeon	Yes/No	✓	✓	✓
Depression, anxiety, and stress	DASS-21	✓	✓	✓
Surgery performed	Self-reported TKJR and arthroscopy		✓	✓
Willingness to undergo surgery	5-point ordinal scale	✓	✓	✓
**Other measures**				
Health economic data	Quality adjusted life years	✓	✓	✓
	Self-reported medication use	✓	✓	✓
	Self-reported use of health services/co-interventions	✓	✓	✓
	Cost-effectiveness ratio		✓	✓
	Work productivity (WHO HPQ Short Form)	✓	✓	✓
Adherence	Number of consultations with physiotherapist^a^		✓	
	Number of consultations with dietitian^b^		✓	
	Duration of consultations with physiotherapist^a^		✓	
	Duration of consultations with dietitian^b^		✓	
	Self-rated adherence to strengthening exercise^a^/ physical activity^a^/weight management^b^, 11-point NRS		✓	
Perceived usefulness	Times accessed website, 5-point ordinal scale		✓	✓
	Usefulness of website, 11-point NRS		✓	
	Usefulness of physiotherapy consultations^a^ 11-point NRS		✓	
	Usefulness of dietitian consultations^b^ 11-point NRS		✓	
	Usefulness of educational resources^a^ 11-point NRS		✓	
	Usefulness of Fitbit^a^ 11-point NRS		✓	
	Usefulness of ketogenic diet^b^ 11-point NRS		✓	
	Usefulness of strengthening exercise program^a^ 11-point NRS		✓	
	Usefulness of physical activity plan^a^ 11-point NRS		✓	
	Video software ease of use^a^ 11-point NRS		✓	
Harms	Adverse events		✓	✓
Determinants	Self-efficacy for symptom control (ASES)	✓	✓	✓
	Self-efficacy for eating (WELQ)	✓	✓	✓
	Attitudes towards self-management (PAM-13)	✓	✓	✓
	Treatment beliefs about arthroplasty (TOA)	✓	✓	✓
	Fear of movement (BFMS)	✓	✓	✓
Long-term surgical rates	Number who have had arthroscopy/arthroplasty		24 months and 60 months	

*NRS* Numeric rating scale, *WOMAC* Western Ontario and McMaster Universities Osteoarthritis Index, *IPEQ-W* Incidental and Planned Exercise Questionnaire, *AQoL-8D* Assessment of Quality of Life Instrument, *DASS* Depression, Anxiety, and Stress Scale, *TKJR* Total knee joint replacement, *WHO HPQ* World Health Organization Health and Work Performance Questionnaire, *ASES* Arthritis Self-efficacy Scale, *WELQ* Weight Efficacy Lifestyle Questionnaire, *PAM* Patient Activation Measure, *TOA* Treatment beliefs in knee and hip OA – arthroplasty subscale, *BFMS* Brief Fear of Movement Scale for osteoarthritis

^a^measured in *Exercise* and *Exercise plus weight management* groups only

^b^measured in *Exercise plus weight management* group only

### Primary outcomes

Primary outcomes are overall average knee pain severity in the last week measured on an 11-point numeric rating scale (NRS), where 0 = ‘no pain’ and 10 = ‘worst pain possible’ [58], and physical function assessed using the physical function subscale of the Western Ontario and McMaster Universities (WOMAC) Osteoarthritis Index [59]. The WOMAC is a disease-specific self-report instrument which has demonstrated validity, reliability, and responsiveness [60]. The physical function subscale contains 17 questions scored from 0 to 4, giving a range of possible scores from 0 (no dysfunction) to 68 (maximum dysfunction).

### Secondary outcomes

Secondary outcome measures include:

i) Self-reported weight, measured in kilograms. Participants are asked to use the same set of scales for these measures across the time points;
ii) Physical activity, assessed using the Incidental and Planned Exercise Questionnaire (IPEQ-W) [61];
iii) Health-related quality of life, measured using the Assessment of Quality of Life Instrument (AQoL-8D) [62];
iv) Global rating of change, scored on a 7-point Likert scale for “Overall change in your study knee since you began the study” from “much worse” to “much better” [63];
v) Satisfaction with care, scored on a 7-point Likert scale for “Overall satisfaction with the care you received in this study” from “extremely unsatisfied” to “extremely satisfied”;
vi) Self-reported appointments with an orthopaedic surgeon;
vii) Depression, anxiety, and stress, measures on the Depression, Anxiety, and Stress Scale (DASS-21) [64];
viii) Self-reported total knee joint replacement and knee arthroscopy surgery;
ix) Willingness to undergo surgery, measured on a 5-point Likert scale ranging from “definitely not willing” to “definitely willing”.

### Descriptive data

At baseline, descriptive data is collected, including: i) age; ii) gender; iii) height and weight; iv) country of birth; v) postal code; vi) Aboriginal or Torres Strait Islander Heritage; vii) education level; viii) current employment status; ix) duration of symptoms; x) length of time since first visit to a doctor for knee pain; xi) previous history of knee arthroscopy and arthroplasty; xii) pain in other parts of the body; xiii) expectation of treatment outcomes, and; xiv) medication use.

### Determinants

i) Self-efficacy for managing arthritis symptoms, measured using the Arthritis Self-Efficacy Scale [65];
ii) Self-efficacy for eating control, measured using the Weight Efficacy Lifestyle Questionnaire [66];
iii) Attitudes towards self-management, measured using the Patient Activation Measure (PAM-13) [67];
iv) Treatment beliefs about arthroplasty, using the Treatment beliefs in knee and hip OsteoArthritis (TOA) questionnaire – Arthroplasty subscale [68];
v) Fear of movement, measured using the Brief Fear of Movement Scale for osteoarthritis [69].

### Additional measures

*Economic data*: assessed by self-reported medication use and healthcare costs (regarding the use of health services), quality-adjusted life years (QALY), cost-effectiveness ratio (calculated using predictions of health care costs and QALYs), and work productivity (using the World Health Organisation Health and Work Performance Questionnaire [70]). Knee surgery costs will be obtained by linking to Medibank data.

*Adverse events*: any undesirable clinical occurrence, whether considered to be treatment-related or not, that includes a clinical sign, symptom, or condition, are self-reported in questionnaires and recorded by the clinician during the consultation. Participants are asked to provide details on the nature of the event, how long it lasted for, and what action they took.

*Adherence*: assessed by recording the number of consultations received from the physiotherapist and/or dietitian. Adherence to prescribed exercise and physical activity programs is self-reported by the participant via questionnaire, being scored on an 11-point NRS (0 = not at all and 10 = completely as instructed). Adherence to the weight management plan, if applicable, is also self-reported and scored on a similar 11-point NRS.

*Perceived usefulness of treatment components*: assessed by self-report with response options ranging from 0 = “not at all” to 10 = “extremely useful”. Usefulness questions relate to the information on the website, consultations with the physiotherapist and/or dietitian, resource booklets, Fitbit, VLCD, strengthening exercise program, and physical activity plan. Participants will also be asked how many times they accessed the website in the last 6-months (5-point scale ranging from “never” to “> 10 times”) and how easy it was for them to use the videoconferencing software (11-point NRS ranging from 0 = “not at all easy” to 10 = “extremely easy”).

*Long-term surgical rates*: via linkage with the data routinely collected by Medibank Private, and will include the number of individuals who had a knee arthroscopy and/or arthroplasty intervention at 2 and 5 years after baseline. These findings will be reported separately from the main trial outcomes.

### Trial sample size

The primary outcomes of this trial include changes in pain (NRS) and physical function (WOMAC) over 6 months and the intervention will be considered beneficial if it changes either one or both of the primary outcomes. A Cochrane Review shows that exercise has a moderate effect size for pain and function in knee OA [10]. Calculations thus assume an effect size between the least-performing treatment group (presumed to be the *Exercise* group) and the control group of 0.4. Given that the effect size between two treatments will be less than between treatment and control, we assumed an effect size of 0.3 between the *Exercise* group and the *Exercise plus weight management* group. We chose 0.3 because any smaller effect is unlikely to be clinically relevant or cost-effective to implement in real-world settings given the considerable expense of the weight management program relative to the exercise components of this combined intervention. Calculations also assume a correlation between baseline and follow-up measurements of 0.4, power 80, 15% loss to follow-up (based on our previous research [71, 72]), and two-sided significance level of 0.05. Based on these calculations we require 173 participants in each treatment group, and 69 participants in the control group arm, giving a total sample size of 415 participants.

There are different levels of clustering for each of the three groups; in the *Exercise* group, participants are clustered by physiotherapist, in the *Exercise plus weight management* group, people are clustered by physiotherapist and dietitian, and in the control group, there is no clustering. Given the geographical and demographic spread of participants, we expect that the intra-cluster correlation of outcomes treated by the same dietitian or physiotherapist to be of the order of 0.01. Modifying the formulae [73] to allow for correlations between baseline and follow-up measurements, and assuming that there are five dietitians delivering the *Exercise plus weight management* treatment, the effect size that can be detected between the *Exercise* and the *Exercise plus weight management* groups with 80% power given the originally calculated sample size increases slightly to 0.32.

### Analysis

We will use intention-to-treat analyses, using multiple imputation to account for missing data and details on the imputation will be reported [74]. Participants will be included in their randomised treatment groups regardless of their post-randomisation behaviour. *P* values less than 0.05 will be considered significant. Outcomes (primary, secondary, and determinants) will be compared between groups as follows: i) *Exercise* vs *Exercise plus weight management*; ii) *Exercise* vs control, and; iii) *Exercise plus weight management* vs control. For continuous data, mean differences in change over time between these groups will be estimated using linear regression models fit to data from both follow-up time points, with random effects for participants, and accounting for clustering by physiotherapist and dietitian in the *Exercise* and *Exercise plus weight management* groups. Models will be adjusted for the stratification variable (history of knee surgery) and values of the outcome at baseline. Terms for time and treatment will be included, with an interaction between the two. Regression assumptions will be assessed using standard diagnostic plots. For binary outcomes, logistic regression models will be fit using generalised estimating equations to account for clustering, with risk differences and 95% confidence intervals calculated. This will include a comparison of the proportion of participants in each group whose improvement meets or exceeds the minimal clinically important difference for each of the primary outcomes. For ordinal outcomes, proportional odds models will be fit similarly. Multinominal models will be applied if the assumption of proportional odds is not valid. To assess the sensitivity of the results for the primary outcomes in situations in which the outcomes are missing not at random, we will apply a pattern-mixture approach that involves fitting regression models that adjust for departures from the missing at random assumption [75].

Economic evaluations will assess and compare the cost-effectiveness of the interventions. This will involve analysis of: i) cost per extra person with a clinically significant improvement in pain (measured as 1.8 point reduction on the pain score) and function (6 unadjusted WOMAC units), and; ii) per quality-adjusted life years (QALYs) gained for the intervention compared to control at 12 months. QALYs will be calculated based on utility scores using the AQoL-8D at baseline and 12 months. The difference in health care usage and productivity lost between baseline and 12 months will be compared for intervention and control groups. The association between utility gains on the AQoL-8D and productivity will also be compared between the intervention and control groups.

To evaluate the longer-term effects of the *Exercise* and *Exercise plus weight management* interventions on total knee joint replacement and knee arthroscopy surgical rates, logistic regression models for surgery will be fit using generalised estimating equations to account for clustering by physiotherapist and dietitian, with treatment effects summarised using risk differences and 95% confidence intervals. The stratifying variable of history of knee surgery will be adjusted for.

To evaluate whether there is a relationship between amount of weight loss and changes in pain and/or function, linear regression models for changes in pain and outcome will be fit and will include a term for weight lost at 6 and 12 months (as percentage of baseline weight), adjusting for baseline level of the outcome, assigned treatment group, and stratifying variables. Demographic variables thought to affect both weight loss and outcomes levels, including age and sex, will also be included in the model. Fractional polynomial terms for weight lost will be assessed to investigate if the relationship between outcomes and weight loss is non-linear. Standard diagnostic plots will be used to assess regression assumptions. We will also evaluate whether there is a relationship between adherence to exercise and changes in pain and/or function by estimating a complier-average causal effect using a two-stage least squares approach [76].

Finally, we will conduct exploratory analyses to investigate whether the relative effects of each intervention on change in each of the primary outcomes is moderated by: i) expectation of treatment effects; ii) sex; iii) pain self-efficacy; iv) BMI; v) employment situation; vi) history of knee surgery (contralateral total knee joint replacement surgery and/or knee arthroscopic surgery); vii) self-efficacy for eating control, and; viii) depression. These moderators were selected based on previous research and/or theoretical plausibility (Table 5). Models will include terms for treatment, the moderator variables, and an interaction between the two. Fractional polynomials will be included for the continuous moderators [88]. The estimated effects of treatment and 95% confidence intervals will be presented for each moderator, with visual representations included for continuous moderators.

**Table 5:** Overview of selected demographic and clinical moderators

Selected moderator variables	Justification		
Expectation of treatment effects	Based on evidence that greater treatment expectations are associated with more favourable outcomes in people with osteoarthritis [77–79]. *Hypothesis*: Participants in the intervention groups who have greater treatment expectations will report greater improvement in primary outcomes than those who have lower treatment expectations (relative to control group).		
Sex	Based on evidence that being male is associated with better outcomes in pain and physical function after supervised strengthening exercises [80] and evidence from a review that being female is associated with greater weight loss intentions [81]. *Hypothesis*: Participants in the intervention groups who are male will report greater improvement in primary outcomes than those who are female (relative to control). Participants in the *Exercise plus weight management* group who are female will report greater improvement in primary outcomes than those who are male (relative to *Exercise* group).		
Pain self-efficacy	Based on evidence that higher self-efficacy associated with better outcomes in pain and quality of life after supervised neuromuscular exercise [82], and greater improvements in pain after and internet-delivered exercise and education program [83]. *Hypothesis*: Participants in the intervention groups who have higher self-efficacy at baseline will report greater improvement in primary outcomes than those who have lower self-efficacy (relative to control group).		
Body mass index	Based on evidence that obesity is associated with better outcomes in quality of life after supervised aquatic exercise [84] and evidence from a review that higher initial BMI is associated with greater weight loss [85]. *Hypothesis*: Participants in the intervention groups who have a higher BMI will report greater improvement in primary outcomes (relative to control) than those who have a lower BMI. Participants in the *Exercise plus weight management* group who have a higher BMI will report greater improvement in primary outcomes than those with a lower BMI (relative to *Exercise* group).		
Employment situation	Based on evidence that being employed associated with greater improvements in pain after an internet-delivered exercise and education program [83]. *Hypothesis*: Participants in the intervention groups who are employed will report greater improvement in primary outcomes than those who are not employed (relative to control group).		
History of knee surgery	Chosen based on theoretical plausibility that knee surgical experience could affect expectations of outcomes and motivation *Hypothesis*: Participants in the intervention groups who have a history of knee surgery will report less improvement in primary outcomes than those without (relative to control group).		
Self-efficacy for eating control	Based on evidence from a review that better control of over-eating and dietary restraint is associated with weight loss and maintenance [86, 87]. *Hypothesis*: Participants in the *Exercise plus weight management* group who have higher self-efficacy for eating control will report greater improvement in primary outcomes than those with lower self-efficacy for eating control (relative to *Exercise* group and to control group).		
Depression	Based on evidence that fewer depressive symptoms is associated with better outcomes in pain and physical function after supervised strengthening exercises [80]. *Hypothesis*: Participants in the intervention groups who have more depressive symptoms at baseline will report less improvement in primary outcomes than those who have less depressive symptoms (relative to control group).		

### Ethics

Ethical approval has been obtained from the University of Melbourne Human Research Ethics Committee (HREC No. 1750443). All participants will provide written informed consent. The funding agency (Medibank) will be responsible for marketing the study to support recruitment, by making the study known (via email, letters, articles, newsletters and social media) to potentially eligible members. All marketing material viewed by member will include a statement that their involvement in the study will not affect their relationship or policy with Medibank. Medibank will not be involved in statistical analysis.

## Discussion

With the rapidly rising burden of knee OA, there is an urgent need for interventions that improve outcomes and that are also widely accessible to people in rural, regional, and metropolitan communities [89]. Although the effectiveness of exercise therapy on pain and function is well-established, there is relatively limited robust evidence about the additional benefits of weight loss in people with knee OA [4].

This trial will be the first to investigate the clinical- and cost-effectiveness of a remotely delivered service combining dietary weight management and exercise, compared to a service involving exercise only and to an online information-only control group. Such a service has the potential to be a low-cost and accessible way in which people with OA can receive care, thus potentially increasing uptake of recommended management methods, like weight loss and exercise. Findings from this study will inform healthcare providers about the effects of incorporating weight loss components into exercise interventions. Moderator analyses using data from this study will also allow us to determine whether specific sub-groups of participants are more likely to respond to these remotely delivered services, which will allow health service providers to better target interventions to those that would benefit most. Qualitative explorations will provide insight into the feasibility and viability of implementing similar weight management programs into private and public healthcare settings from the perspective of both the participants receiving care, as well as the clinicians involved in delivering it.

A potential limitation of this trial is the targeted sample of participants, including only those who are Medibank Private members with a level of membership that includes arthroplasty surgery. This may potentially limit the generalisability of our findings, however, Medibank Private is one is one of the largest providers of private health insurance in Australia, and participants will be recruited Australia-wide to attempt to maximise the generalisability of the cohort.

## Data Availability

The datasets used and/or analysed during the current study will be available from the corresponding author (k.bennell@unimelb.edu.au) on reasonable request once the study has been completed.

## Abbreviations

BMI: Body mass index
CSIRO: Commonwealth Scientific and Industrial Research Organisation
MITI: Motivational Interviewing Treatment Integrity tool
NRS: Numeric rating scale
NSAIDS: Non-steroidal anti-inflammatory drugs
OA: Osteoarthritis
RCT: Randomised controlled trial
RPE: Rating of perceived exertion
VLCD: Very low calorie diet
WOMAC: Western Ontario and McMaster Universities Osteoarthritis Index
